# Computation of the basic reproduction numbers for reaction-diffusion epidemic models

**DOI:** 10.3934/mbe.2023680

**Published:** 2023-07-19

**Authors:** Chayu Yang, Jin Wang

**Affiliations:** 1 Department of Mathematics, University of Nebraska-Lincoln, 1400 R Street, Lincoln, NE 68588, USA; 2 Department of Mathematics, University of Tennessee at Chattanooga, 615 McCallie Avenue, Chattanooga, TN 37403, USA

**Keywords:** reaction-diffusion systems, numerical calculation, matrix analysis

## Abstract

We consider a class of k-dimensional reaction-diffusion epidemic models (k=1,2,⋯) that are developed from autonomous ODE systems. We present a computational approach for the calculation and analysis of their basic reproduction numbers. Particularly, we apply matrix theory to study the relationship between the basic reproduction numbers of the PDE models and those of their underlying ODE models. We show that the basic reproduction numbers are the same for these PDE models and their associated ODE models in several important scenarios. We additionally provide two numerical examples to verify our analytical results.

## 1. Introduction

Partial differential equations (PDEs) of the reaction-diffusion type are extensively used in the modeling of infectious diseases [1–8]. The focus of the present paper is to study the basic reproduction numbers for a class of reaction-diffusion epidemic models constructed from autonomous systems of ordinary differential equations (ODEs). The underlying ODE models represent the dynamics of disease transmission and spread that are spatially homogeneous, whereas the PDE models, with diffusion terms added, emphasize the movement and dispersal of the hosts and pathogens over a (typically heterogeneous) spatial domain.

The basic reproduction number, commonly denoted by ${ℛ}_{0}$, is a critical quantity to quantify the transmission risk of an infectious disease. It measures the expected number of secondary infections produced by one infective individual in a completely susceptible population. ${ℛ}_{0}$ is often used to characterize the threshold behavior of an epidemic, with disease eradication if ${ℛ}_{0}<1$ and disease persistence if ${ℛ}_{0}>1$. The theory of the basic reproduction numbers for ODE-based autonomous epidemic models is well developed, and the calculation of ${ℛ}_{0}$ follows a standard procedure based on the next-generation matrix technique [9, 10].

Many efforts have been devoted to the definition, calculation, and analysis of the basic reproduction numbers for PDE models, such as reaction-diffusion epidemic systems, which are more complex than their ODE counterparts. Thieme [11] introduced a theoretical framework to study ${ℛ}_{0}$, defined as the spectral radius of a resolvent-positive operator, of such reaction-diffusion equations. Using the theory of principal eigenvalues, Wang and Zhao [12] defined ${ℛ}_{0}$ as the spectral radius of a next-infection operator for a general class of reaction-diffusion models. They showed that the ${ℛ}_{0}$ of such a PDE system is the same as that of the underlying ODE system when the diffusion rates are positive constants and the next-generation matrices are independent of the spatial location. Asymptotic profiles of ${ℛ}_{0}$ for reaction-diffusion epidemic systems with constant diffusion rates were investigated in [13–16]. Particularly, Chen and Shi [14] showed that ${ℛ}_{0}$ approaches the spectral radius of a spatially averaged next-generation matrix when the diffusion rates tend to infinity, and ${ℛ}_{0}$ approaches the maximum value of a local reproduction number when the diffusion rates tend to zero. Moreover, reproduction numbers for time-periodic reaction-diffusion systems were discussed in [17, 18]. Other theoretical studies related to the reaction-diffusion epidemic models and their basic reproduction numbers include [1, 6, 8, 19–23].

The goal of the present work is twofold: (1) to produce practically useful means to compute the basic reproduction numbers of reaction-diffusion epidemic models, since the classical next-generation matrix technique for autonomous ODE systems is no longer applicable; and (2) to gain a deeper understanding of the relationship between the basic reproduction number of a reaction-diffusion PDE model, ${ℛ}_{0}^{\text{PDE}}$, and that of its underlying ODE model, ${ℛ}_{0}^{\text{ODE}}$. To that end, we will focus on a class of reaction-diffusion epidemic systems that are developed by adding diffusion terms to autonomous ODE systems, where the diffusion rates generally are functions of the location variables representing the spatial heterogeneity. In a prior study [24], we proposed a numerical method to compute the value of ${ℛ}_{0}$ for such reaction-diffusion epidemic models on one-dimensional (1D) spatial domains. The essential idea is the reduction of the infinite-dimensional operator eigenvalue problem for a PDE system to a finite-dimensional matrix eigenvalue problem.

In the present study, we will make a nontrivial extension of the methodology in [24] to reaction-diffusion epidemic systems on k-dimensional spatial domains, where k≥1 can be any positive integer. Such an extension, in addition to making the theory and methodology more complete, would facilitate the study of more practical applications where these PDE models are utilized to investigate the transmission and spread of infectious diseases in the real world. Based on the numerical formulation, we will use matrix theory to analyze the relationship between the PDE-based ${ℛ}_{0}^{\text{PDE}}$ and the ODE-based ${ℛ}_{0}^{\text{ODE}}$. The matrix analysis involved in the current work for k-dimensional models is significantly harder than that for one-dimensional models. We will show that under several important scenarios, such as the presence of a single infected compartment, constant diffusion rates, uniform diffusion of the infected compartments, and partial diffusion in a system, the two basic reproduction numbers equal each other.

We organize the remainder of this paper as follows. In Section 2, we present the k-dimensional reaction-diffusion epidemic system and the definition of its basic reproduction number. In Section 3, we describe the details of our computational method for ${ℛ}_{0}^{\text{PDE}}$ and then analyze the relationship between ${ℛ}_{0}^{\text{PDE}}$ and ${ℛ}_{0}^{\text{ODE}}$. In Section 4, we provide specific numerical examples to verify our analytical findings. Finally, we conclude the paper with some discussion in Section 5.

## 2. Reaction-diffusion epidemic model

Let n be a positive integer and $U\left(t,x\right)={\left(u_{1}\left(t,x\right),\ldots ,u_{n}\left(t,x\right)\right)}^{T}$ be a vector-valued function that represents the hosts and pathogens related to an infectious disease, with each $u_{i}\left(t,x\right)$ denoting the density of the (host or pathogen) population in compartment i(1≤i≤n) at time t and location x. We are concerned with the k-dimensional spatial domain ${\left[0,1\right]}^{k}$, where k≥1 is an integer. We consider the following reaction-diffusion epidemic system

(2.1) $$\{\begin{array}{l} \frac{\partial u_{i}}{\partial t}=\nabla \cdot \left(d_{i}\left(x\right)\nabla u_{i}\right)+{ℱ}_{i}\left(U\right)-{𝒱}_{i}\left(U\right),\;1\leq i\leq n,t>0,\;x\in {\left[0,1\right]}^{k};\\ \frac{\partial u_{i}}{\partial v}=0,\;1\leq i\leq n,\;t>0,\;x\in \partial {\left[0,1\right]}^{k}, \end{array}$$

with appropriate initial conditions. In this model, $d_{i}\left(x\right)\left(1\leq i\leq n\right)$ denotes the diffusion rate at location x and is assumed to be continuously differentiable on ${\left[0,1\right]}^{k}$. ${ℱ}_{i}\left(U\right)$ denotes the rate of generation for newly infected individuals in compartment i, and ${𝒱}_{i}\left(U\right)={𝒱}_{i}^{-}\left(U\right)-{𝒱}_{i}^{+}\left(U\right)$, with ${𝒱}_{i}^{+}$ denoting the transfer rate of individuals into compartment i and ${𝒱}_{i}^{-}$ the transfer rate of individuals out of compartment i. Note that ${ℱ}_{i}\left(U\right)$ and ${𝒱}_{i}\left(U\right),i=1,2,\cdots ,n$, are functions of U only, so that the PDE model (2.1) is associated with an underlying ODE model, discussed in Appendix A. In addition, v is the unit normal vector on the boundary $\partial {\left[0,1\right]}^{k}$.

Without loss of generality, we assume that $U_{I}=U_{I}\left(t,x\right)={\left(u_{1},\ldots ,u_{m}\right)}^{T}$ denotes all the infected compartments in the vector U, where 1≤m<n. Consequently, the set of all disease-free steady states is defined as $U_{s}=\left\{U\geq 0:u_{i}=0,i=1,\ldots ,m\right\}$.

System (2.1) can be re-written as

(2.2) $$\{\begin{array}{l} \frac{\partial u_{i}}{\partial t}=d_{i}\left(x\right)\Delta u_{i}-c_{i}\left(x\right)\cdot \nabla u_{i}+{ℱ}_{i}\left(U\right)-{𝒱}_{i}\left(U\right),\;1\leq i\leq n,t>0,\;x\in {\left[0,1\right]}^{k};\\ \frac{\partial u_{i}}{\partial v}=0,\;1\leq i\leq n,t>0,\;x\in \partial {\left[0,1\right]}^{k}, \end{array}$$

where

(2.3) $$c_{i}\left(x\right)=\left(c_{i1}\left(x\right),c_{i2}\left(x\right),\;\ldots ,c_{ik}\left(x\right)\right)=-\nabla d_{i}\left(x\right),\;1\leq i\leq n.$$

In what follows, we investigate the PDE system (2.2), where our results can be easily applied to the original system (2.1) under the condition (2.3).

Following the theoretical framework in [12], we let T(t) be the solution semigroup on $C\left({\left[0,1\right]}^{k},{\mathbb{R}}^{m}\right)$ associated with the following linear reaction-diffusion equation:

(2.4) $$\{\begin{array}{l} \frac{\partial u_{i}}{\partial t}=d_{i}\left(x\right)\Delta u_{i}-c_{i}\left(x\right)\cdot \nabla u_{i}-{𝒱}_{i}\left(U\right),\;1\leq i\leq m,\;t>0,\;x\in {\left[0,1\right]}^{k};\\ \frac{\partial u_{i}}{\partial v}=0,\;1\leq i\leq m,\;t>0,\;x\in \partial {\left[0,1\right]}^{k}. \end{array}$$

Let the distribution of the initial infections, i.e. $U_{I}\left(0,x\right)$, be $U_{m}\left(x\right)={\left(u_{1}\left(x\right),\ldots ,u_{m}\left(x\right)\right)}^{T}$. Then the distribution of these infections after time t>0 is given by $T\left(t\right)\left(U_{m}\left(x\right)\right)$. Let F be the generation matrix of new infections (see Appendix A). Then the distribution of new infections at any time t>0 is $FT\left(t\right)\left(U_{m}\left(x\right)\right)$ and the distribution of the total new infections is represented by $\int_{0}^{+\infty }FT\left(t\right)\left(U_{m}\left(x\right)\right)dt$. Therefore, the next-generation operator L, which maps the distribution of initial infections to the distribution of the total infective individuals generated during the infectious period, is defined by

(2.5) $$L:\;U_{m}\left(x\right)\mapsto \int_{0}^{+\infty }FT\left(t\right)\left(U_{m}\left(x\right)\right)dt.$$

Consequently, the basic reproduction number for the PDE system (2.2) is the spectral radius of the operator L :

(2.6) $${ℛ}_{0}^{\text{PDE}}=\rho \left(L\right).$$

Meanwhile, we introduce the operator $\Gamma :{\mathbb{R}}^{m}\mapsto {\mathbb{R}}^{m}$ by

(2.7) $$\Gamma =D_{m}\left(x\right)\Delta -C_{m}\left(x\right)\cdot \nabla -V,$$

where, for $U={\left(u_{1},\;\cdots \;,u_{m}\right)}^{T}\in {\mathbb{R}}^{m},\left(D_{m}\left(x\right)\Delta \right)U={\left(d_{1}\left(x\right)\Delta u_{1},\;\ldots ,d_{m}\left(x\right)\Delta u_{m}\right)}^{T}$ and $\left(C_{m}\left(x\right)\cdot \nabla \right)U={\left(c_{1}\left(x\right)\cdot \nabla u_{1},\;\ldots ,c_{m}\left(x\right)\cdot \nabla u_{m}\right)}^{T}$, with $c_{j}\left(x\right)=\left(c_{j1}\left(x\right),c_{j2}\left(x\right),\;\ldots ,c_{jk}\left(x\right)\right),1\leq j\leq m$. Then we can obtain an essential characterization of the next-infection operator,

(2.8) $$L=-F\Gamma^{-1},$$

with details provided in Appendix B.

Below we focus our attention on the numerical calculation of ${ℛ}_{0}^{\text{PDE}}$, based on Eqs (2.6) and (2.8), and the analysis of its relationship with the basic reproduction number ${ℛ}_{0}^{\text{ODE}}$ of the underlying ODE model (A.1). Throughout our discussion, we assume that the conditions (A1)–(A4) in Appendix A and (B1)–(B3) in Appendix B hold.

## 3. Numerical formulation and matrix analysis

Let λ be an eigenvalue of the operator L such that L(ϕ(x))=λϕ(x) for an eigenvector $\phi \left(x\right)={\left(\phi_{1}\left(x\right),\ldots ,\phi_{m}\left(x\right)\right)}^{T}$. Then from Eq (2.8) we have

(3.1) $$-F\Gamma^{-1}\left(\phi \left(x\right)\right)=\lambda \phi \left(x\right).$$

Let $\psi \left(x\right)=-\Gamma^{-1}\left(\phi \left(x\right)\right)$, whe $\psi \left(x\right)={\left(\psi_{1}\left(x\right),\ldots ,\psi_{m}\left(x\right)\right)}^{T}$. . Then −Γ(ψ(x))=ϕ(x). . Based on the condition (B3), this equation can be written as

(3.2) $$-\left(d_{p}\left(x\right)\Delta \psi_{p}\left(x\right)-c_{p}\left(x\right)\cdot \nabla \psi_{p}\left(x\right)-v_{p}\psi_{p}\left(x\right)\right)=\phi_{p}\left(x\right),\;1\leq p\leq m.$$

Pick a sufficiently large integer N>0 and denote

$$\begin{array}{ll} d_{p}^{j_{1}\ldots j_{k}}=d_{p}\left(\frac{j_{1}}{N},\frac{j_{2}}{N},\;\ldots ,\frac{j_{k}}{N}\right), & c_{pj}^{j_{1}\ldots j_{k}}=c_{pr}\left(\frac{j_{1}}{N},\frac{j_{2}}{N},\;\ldots ,\frac{j_{k}}{N}\right),\\ \psi_{p}^{j_{1}\ldots j_{k}}=\psi_{p}\left(\frac{j_{1}}{N},\frac{j_{2}}{N},\;\ldots ,\frac{j_{k}}{N}\right), & \phi_{p}^{j_{1}\ldots j_{k}}=\phi_{p}\left(\frac{j_{1}}{N},\frac{j_{2}}{N},\;\ldots ,\frac{j_{k}}{N}\right), \end{array}$$

for any integers $0\leq j_{1},j_{2},\;\ldots ,j_{k}\leq N$ and 1≤r≤k. Apply the standard centered difference scheme to Eq (3.2) on the spatial domain ${\left[0,1\right]}^{k}$. Then for any $0\leq j_{1},\;\ldots ,j_{k}\leq N$, we obtain

(3.3) $$-d_{p}^{j_{1}\ldots j_{k}}\overset{k}{\underset{r=1}{\sum }}\frac{\psi_{p}^{j_{1}\ldots \left(j_{r}+1\right)\ldots j_{k}}-2\psi_{p}^{j_{1}\ldots j_{k}}+\psi_{p}^{j_{1}\ldots \left(j_{r}-1\right)\ldots j_{k}}}{1/N^{2}}+\overset{k}{\underset{r=1}{\sum }}c_{pr}^{j_{1}\ldots j_{k}}\frac{\psi_{p}^{j_{1}\ldots \left(j_{r}+1\right)\ldots j_{k}}-\psi_{p}^{j_{1}\ldots \left(j_{r}-1\right)\ldots j_{k}}}{2/N}+v_{p}\psi_{p}^{j_{1}\ldots j_{k}}\approx \phi_{i}^{j_{1}\ldots j_{k}},$$

and $\psi_{p}^{j_{1}\ldots \left(-1\right)\ldots j_{k}}=\psi_{p}^{j_{1}\ldots \left(1\right)\ldots j_{k}},\psi_{p}^{j_{1}\ldots \left(N+1\right)\ldots j_{k}}=\psi_{p}^{j_{1}\ldots \left(N-1\right)\ldots j_{k}}$, where (−1),(1),(N+1), and (N−1) are all in the $\left(j_{r}\right)$ position inside the permutation $j_{1}\ldots j_{k}$ for any 1≤r≤k by the Neumann boundary condition. Next, we can write the above ${\left(N+1\right)}^{k}$ approximate equations of (3.3) in the following matrix form

(3.4) $$A_{p}\Psi_{p}\approx \Phi_{p},$$

where $A_{p}=\left(a_{ij}^{\left(p\right)}\right)$ is a ${\left(N+1\right)}^{k}\times {\left(N+1\right)}^{k}$ matrix, $1\leq i,j\leq {\left(N+1\right)}^{k}$, and

$$\Psi_{p}={\left(\psi_{p}^{0\ldots 00},\;\ldots ,\psi_{p}^{0\ldots 0N},\;\ldots ,\psi_{p}^{0\ldots N\ldots 0},\;\ldots ,\psi_{p}^{0\ldots N\ldots N},\;\ldots ,\psi_{p}^{N\ldots N0},\;\ldots ,\psi_{p}^{N\ldots NN}\right)}^{T},\;\Phi_{p}={\left(\phi_{p}^{0\ldots 00},\;\ldots ,\phi_{p}^{0\ldots 0N},\;\ldots ,\phi_{p}^{0\ldots N\ldots 0},\;\ldots ,\phi_{p}^{0\ldots N\ldots N},\;\ldots ,\phi_{p}^{N\ldots .N0},\;\ldots ,\phi_{p}^{N\ldots NN}\right)}^{T}.$$

Note that for any $0\leq j_{1},\;\ldots ,j_{k}\leq N$, the coefficient of $\psi_{p}^{j_{1}\ldots j_{k}}$ in Eq (3.3) is a diagonal entry of $A_{p}$, which is equal to a positive number $2kN^{2}d_{p}^{j_{1}\ldots j_{k}}+v_{p}$. Define

$$N_{0}=\underset{1\leq p\leq m}{\text{max}}\left\{N_{p}\right\},\text{ where }N_{p}=\text{max}\left\{\frac{{\left\|c_{pr}\left(x\right)\right\|}_{\infty }}{2d_{0}},\;1\leq r\leq k\right\},$$

and where $d_{0}$ is a positive lower bound for the diffusion rates (see condition (B2)). Then for $N>N_{0}$, the off-diagonal entries $-N^{2}d_{p}^{j_{1}\ldots j_{k}}+\frac{1}{2}Nc_{pr}^{j_{1}\ldots j_{k}}$ and $-N^{2}d_{p}^{j_{1}\ldots j_{k}}-\frac{1}{2}Nc_{pr}^{j_{1}\ldots j_{k}}$ are nonpositive for all 1≤r≤k. Hence for any eigenvalue λ of $A_{p}$, by the Gershgorin Circle Theorem, there exists $1\leq i\leq {\left(N+1\right)}^{k}$ such that

$$\left|\lambda -a_{ii}^{\left(p\right)}\right|\leq \underset{j\neq i}{\sum }\left|a_{ij}^{\left(p\right)}\right|=\left|a_{ii}^{\left(p\right)}-v_{p}\right|.$$

Thus, $\text{Re}\left(\lambda \right)\geq v_{p}>0$ and $A_{p}$ is invertible. Moreover, we have $\rho \left(A_{p}^{-1}\right)\leq 1/\text{Re}\left(\lambda \right)\leq 1/v_{p},1\leq p\leq m$. This leads to the following lemma.

**Lemma 3.1.** *Let* $N>N_{0}$ *and* $\lambda_{A_{p}}$ *be an eigenvalue of matrix* $A_{p}$. *Then, for* 1≤p≤m, *the real part of* $\lambda_{A_{p}}$ *satisfies* $Re\left(\lambda_{A_{p}}\right)\geq v_{p}$, *and, consequently,* $A_{p}$ *is invertible*.

In addition, for any $0\leq j_{1},\ldots ,j_{k}\leq N$, if we fix $\psi_{p}^{j_{1}\ldots \left(j_{r}+1\right)\ldots j_{k}}=\psi_{p}^{j_{1}\ldots j_{k}}=\psi_{p}^{j_{1}\ldots \left(j_{r}-1\right)\ldots j_{k}}=1$ for all 1≤r≤k in Eq (3.3), then the left-hand side of Eq (3.3) is the sum of the $\left(1+\sum_{r=1}^{k}j_{r}{\left(N+1\right)}^{k-r}\right)$-th row of the matrix $A_{p}$, which is obviously equal to $v_{p}$. Hence the sum of each row of $A_{p}$ is $v_{p}$, which implies $v_{p}$ is an eigenvalue of $A_{p}$, and consequently, $1/v_{p}$ is an eigenvalue of $A_{p}^{-1}$. Therefore $\rho \left(A_{p}^{-1}\right)=1/v_{p}$. We obtain the following result.

**Lemma 3.2.** *For all* $N>N_{0}$, *we have* $\rho \left(A_{p}^{-1}\right)=1/v_{p},1\leq p\leq m$.

Denote $\Psi ={\left(\Psi_{1}^{T},\ldots ,\Psi_{m}^{T}\right)}^{T},\Phi ={\left(\Phi_{1}^{T},\ldots ,\Phi_{m}^{T}\right)}^{T}$, and

$$A=\text{diag}\left(A_{1},\ldots ,A_{m}\right).$$

Then A is invertible and $\Psi \approx A^{-1}\Phi$ by Eq (3.4). It follows from Eq (3.1) that

(3.5) $$F\psi \left(x\right)=-F\Gamma^{-1}\left(\phi \left(x\right)\right)=\lambda \phi \left(x\right),$$

which yields

(3.6) $$\left(F⊗I_{{\left(N+1\right)}^{k}}\right)\Psi =\lambda \Phi$$

for any integer N>0, where $I_{{\left(N+1\right)}^{k}}$ is the ${\left(N+1\right)}^{k}\times {\left(N+1\right)}^{k}$ identity matrix and ⊗ denotes the Kronecker product that is defined as follows: for any r×s matrix $M=\left(m_{ij}\right)$ and p×q matrix Q,

$$M⊗Q=\left[\begin{array}{ccc} m_{11}Q & \cdots & m_{1s}Q\\ \vdots & \ddots & \vdots \\ m_{r1}Q & \cdots & m_{rs}Q \end{array}\right].$$

With the substitution of $\Psi \approx A^{-1}\Phi$ into Eq (3.6), our numerical formulation leads to

(3.7) $$\left(F⊗I_{{\left(N+1\right)}^{k}}\right)A^{-1}\Phi \approx \lambda \Phi .$$

From the basic theory of finite difference schemes [25,26], the solution of Eq (3.7) (or, equivalently, Eq (3.3)) converges to the solution of Eq (3.1) (or, equivalently, Eq (3.2)) when N→∞. Hence, for any ε>0, we can pick N sufficiently large such that

$$\left|\rho \left(\left(F⊗I_{{\left(N+1\right)}^{k}}\right)A^{-1}\right)-\rho \left(L\right)\right|<\varepsilon .$$

Letting ε→0, we obtain our central result for the computation of ${ℛ}_{0}^{\text{PDE}}$ :

(3.8) $${ℛ}_{0}^{\text{PDE}}=\underset{N\to \infty }{\text{lim}}\rho \left(\left(F⊗I_{{\left(N+1\right)}^{k}}\right)A^{-1}\right).$$

We have reduced the original operator eigenvalue problem (3.1) to a matrix eigenvalue problem (3.7). Since there are many efficient numerical techniques available for computing eigenvalues of matrices [27,28], our method facilitates practical evaluation of the basic reproduction number for such a reaction-diffusion epidemic model.

Additionally, our numerical formulation provides important insight into the property of ${ℛ}_{0}^{\text{PDE}}$. In what follows, we apply matrix theory to conduct an analysis of ${ℛ}_{0}^{\text{PDE}}$ and its connection to ${ℛ}_{0}^{\text{ODE}}$, based on Eq (3.8). We first introduce the following lemma.

**Lemma 3.3.** *Assume that $X=\left(x_{ij}\right)$ is an m×m matrix and $Y_{ij}\left(1\leq i,j\leq m\right)$ are n×n matrices. If there exists a nonsingular matrix P such that $P^{-1}Y_{ij}P=U_{ij}$ for all i,j=1, …,m, where $U_{ij}$ is an upper triangular matrix with diagonal entries $y_{ij}^{\left(1\right)},\;\ldots ,y_{ij}^{\left(n\right)}$, then*

$$\text{det}\left[\begin{array}{ccc} x_{11}Y_{11} & \cdots & x_{1m}Y_{1m}\\ \vdots & \vdots & \vdots \\ x_{m1}Y_{m1} & \cdots & x_{mm}Y_{mm} \end{array}\right]=\overset{n}{\underset{k=1}{\prod }}\text{det}\left[\begin{array}{ccc} x_{11}y_{11}^{\left(k\right)} & \cdots & x_{1m}y_{1m}^{\left(k\right)}\\ \vdots & \vdots & \vdots \\ x_{m1}y_{m1}^{\left(k\right)} & \cdots & x_{mm}y_{mm}^{\left(k\right)} \end{array}\right].$$

The proof of Lemma 3.3 is similar to that of Lemma 4.2 in [24]. Now we state our main results regarding the relationship between ${ℛ}_{0}^{\text{PDE}}$ and ${ℛ}_{0}^{\text{ODE}}$ in the following three theorems.

**Theorem 3.1.** *(1) In general, we have* ${ℛ}_{0}^{PDE}\geq {ℛ}_{0}^{ODE}$.

(2) If F is a triangular matrix, then ${ℛ}_{0}^{PDE}={ℛ}_{0}^{ODE}$. Particularly, if m=1, then ${ℛ}_{0}^{PDE}={ℛ}_{0}^{ODE}$.

*Proof*. (1) Let $\text{e}={\left(1,1\ldots ,1\right)}^{\text{T}}$ be a vector with all the ${\left(N+1\right)}^{k}-1$ entries being 1 ‘s, and let $\text{P}=\left[\begin{array}{cc} 1 & 0\\ e & I_{{\left(N+1\right)}^{k}-1} \end{array}\right]$. Since the sum of each row of $A_{i}$ is $v_{i}$, we have

$$P^{-1}A_{i}^{-1}P=\left[\begin{array}{cc} 1/v_{i} & \alpha_{i}^{T}\\ 0 & S_{i} \end{array}\right],$$

where $\alpha_{i}$ is a $\left[{\left(N+1\right)}^{k}-1\right]$-dimensional vector and $S_{i}$ is a $\left[{\left(N+1\right)}^{k}-1\right]\times \left[{\left(N+1\right)}^{k}-1\right]$ matrix with ${\left(N+1\right)}^{k}-1$ rows and ${\left(N+1\right)}^{k}-1$ columns. Similar to the proof of Lemma 4.2 in [24], we obtain that $\text{det}\left(\lambda I_{m}-FV^{-1}\right)$ is a factor of $\text{det}\left[\lambda I_{m{\left(N+1\right)}^{k}}-\left(F⊗I_{{\left(N+1\right)}^{k}}\right)A^{-1}\right]$; that is, each eigenvalue of $FV^{-1}$ is an eigenvalue of $\left(F⊗I_{{\left(N+1\right)}^{k}}\right)A^{-1}$. Thus, $\rho \left(\left(F⊗I_{{\left(N+1\right)}^{k}}\right)A^{-1}\right)\geq \rho \left(FV^{-1}\right)$. Letting N→∞, we obtain ${ℛ}_{0}^{\text{PDE}}\geq {ℛ}_{0}^{\text{ODE}}$.

(2) This statement directly follows from Lemma 3.2, since

$$\rho \left(\left(F⊗I_{{\left(N+1\right)}^{k}}\right)A^{-1}\right)=\underset{1\leq i\leq m}{\text{max}}\left\{\rho \left(F_{ii}A_{i}^{-1}\right)\right\}=\underset{1\leq i\leq m}{\text{max}}\left\{F_{ii}/v_{i}\right\}=\rho \left(FV^{-1}\right).$$

**Theorem 3.2.** *If the matrix set* ${\left\{A_{i}\right\}}_{i=1}^{m}$ *for system* (2.2) *is a commuting family where each pair of matrices commute with each other, then* ${ℛ}_{0}^{PDE}={ℛ}_{0}^{ODE}$.

*Proof*. By Theorem 3.1(1), it suffices to show that ${ℛ}_{0}^{\text{PDE}}\leq {ℛ}_{0}^{\text{ODE}}$. Since ${\left\{A_{i}\right\}}_{i=1}^{m}$ is a commuting family of matrices, then ${\left\{A_{i}^{-1}\right\}}_{i=1}^{m}$ is a commuting family. Hence, there exists a nonsingular matrix Q such that

$$QA_{i}^{-1}Q^{-1}=B_{i},$$

where $B_{i}$ is an upper triangular matrix with diagonal elements $\alpha_{i1},\;\ldots ,\alpha_{i{\left(N+1\right)}^{k}}$ and $\left|\alpha_{ij}\right|\leq 1/v_{i}$ for $i=1,\;\ldots ,m;j=1,\;\ldots ,{\left(N+1\right)}^{k}$. Consequently, by Lemma 3.3, we have

(3.9) $$\text{det}\left(\lambda I_{m{\left(N+1\right)}^{k}}-\left(F⊗I_{{\left(N+1\right)}^{k}}\right)A^{-1}\right)=\overset{{\left(N+1\right)}^{k}}{\underset{j=1}{\prod }}\text{det}\left(\lambda I_{m}-O_{j}\right),$$

where $O_{j}=\left[\begin{array}{ccc} F_{11}\alpha_{1j} & \cdots & F_{1m}\alpha_{mj}\\ \vdots & \vdots & \vdots \\ F_{m1}\alpha_{1j} & \cdots & F_{mm}\alpha_{mj} \end{array}\right]$ for $1\leq j\leq {\left(N+1\right)}^{k}$. Thus, Eq (3.9) indicates that

(3.10) $$\rho \left(\left(F⊗I_{{\left(N+1\right)}^{k}}\right)A^{-1}\right)=\underset{1\leq j\leq {\left(N+1\right)}^{k}}{\text{max}}\left\{\rho \left(O_{j}\right)\right\}.$$

Since F is nonnegative by assumptions (A1) and (A4), then $\left|O_{j}\right|\leq FV^{-1}$, where $\left|O_{j}\right|=\left[\begin{array}{ccc} \left|F_{11}\alpha_{1j}\right| & \cdots & \left|F_{1m}\alpha_{mj}\right|\\ \vdots & \vdots & \vdots \\ \left|F_{m1}\alpha_{1j}\right| & \cdots & \left|F_{mm}\alpha_{mj}\right| \end{array}\right]$ We thus obtain $\rho \left(O_{j}\right)\leq \rho \left(\left|O_{j}\right|\right)\leq \rho \left(FV^{-1}\right)$. Therefore, $\rho \left(\left(F⊗I_{{\left(N+1\right)}^{k}}\right)A^{-1}\right)\leq \rho \left(FV^{-1}\right)$, which implies ${ℛ}_{0}^{\text{PDE}}\leq {ℛ}_{0}^{\text{ODE}}$.

Next, we provide sufficient and necessary conditions to characterize the scenarios where ${\left\{A_{p}\right\}}_{p=1}^{m}$ is a commuting family.

**Theorem 3.3.** *For any integer* $N>N_{0}$, *the matrix set* ${\left\{A_{p}\right\}}_{p=1}^{m}$ *associated with system* (2.2) *is a commuting family if and only if there exist constants* $\delta_{p},\sigma_{pr}$, *and continuous functions* $d\left(x\right),g_{r}\left(x\right),1\leq p\leq m,1\leq r\leq k$, *such that*

$$d_{p}\left(x\right)=\delta_{p}d\left(x\right),\;c_{pr}\left(x\right)=\sigma_{pr}g_{r}\left(x\right)$$

*and*

$$\delta_{p}\sigma_{qr}=\delta_{q}\sigma_{pr},\;\sigma_{pi}\sigma_{qj}=\sigma_{qi}\sigma_{pj}$$

*for any* 1≤p,q≤m *and* 1≤r,i,j≤k.

*Proof*. For any $0\leq j_{1},\;\ldots ,j_{k}\leq N$, we denote the $\left(1+\sum_{r=1}^{k}j_{r}{\left(N+1\right)}^{k-r}\right)$-th entry of a ${\left(N+1\right)}^{k}-$ dimensional vector β by ${\left(\beta \right)}_{j_{1}\ldots j_{k}}$ and write $A_{p}=N^{2}H_{p}+\frac{N}{2}G_{p}+v_{p}I_{{\left(N+1\right)}^{k}}$ for p=1, …,m, where $H_{p}$ satisfies

$${\left(H_{p}\Psi_{p}\right)}_{j_{1}\ldots j_{k}}=-d_{p}^{j_{1}\ldots j_{k}}\overset{k}{\underset{r=1}{\sum }}\left(\psi_{p}^{j_{1}\ldots \left(j_{r}+1\right)\ldots j_{k}}-2\psi_{p}^{j_{1}\ldots j_{k}}+\psi_{p}^{j_{1}\ldots \left(j_{r}-1\right)\ldots j_{k}}\right)$$

and $G_{p}$ satisfies

$${\left(G_{p}\Psi_{p}\right)}_{j_{1}\ldots j_{k}}=\overset{k}{\underset{r=1}{\sum }}c_{pr}^{j_{1}\ldots j_{k}}\left(\psi_{p}^{j_{1}\ldots \left(j_{r}+1\right)\ldots j_{k}}+\psi_{p}^{j_{1}\ldots \left(j_{r}-1\right)\ldots j_{k}}\right).$$

Thus, for any 1≤p,q≤m, the equality

$$A_{p}A_{q}=A_{q}A_{p}$$

is equivalent to

(3.11) $$N^{2}\left(H_{p}H_{q}-H_{q}H_{p}\right)+\frac{N}{2}\left(H_{p}G_{q}+G_{p}H_{q}-H_{q}G_{p}-G_{q}H_{p}\right)+\frac{1}{4}\left(G_{p}G_{q}-G_{q}G_{p}\right)=0.$$

Since Eq (3.11) holds for any $N>N_{0}$, we can conclude that

$$H_{p}H_{q}=H_{q}H_{p},\;H_{p}G_{q}+G_{p}H_{q}=H_{q}G_{p}+G_{q}H_{p},\;G_{p}G_{q}=G_{q}G_{p},$$

for any 1≤p,q≤m. Following similar algebraic manipulations as those in [24], we can conclude the following: (i) $H_{p}H_{q}=H_{q}H_{p}$ implies that there exist constants $\delta_{p},1\leq p\leq m$, and a continuous function d(x) such that

$$d_{p}\left(x\right)=\delta_{p}d\left(x\right)$$

(ii) $G_{p}G_{q}=G_{q}G_{p}$ implies that there exist constants $\sigma_{pr},1\leq p\leq m,1\leq r\leq k$, and continuous functions $g_{r}\left(x\right),1\leq r\leq k$, such that

$$c_{pr}\left(x\right)=\sigma_{pr}g_{r}\left(x\right),\;\sigma_{pi}\sigma_{qj}=\sigma_{qi}\sigma_{pj}$$

for any 1≤p,q≤m,1≤r,i,j≤k. Substitute functions $d_{p}\left(x\right)=\delta_{p}d\left(x\right),c_{pr}\left(x\right)=\sigma_{pr}g_{r}\left(x\right)$ into matrices $H_{p},H_{q},G_{p}$ and $G_{q}$. Then for any 1≤p,q≤m, we can obtain that $H_{p}G_{q}+G_{p}H_{q}=H_{q}G_{p}+G_{q}H_{p}$ holds if and only if

$$\delta_{p}\sigma_{qr}=\delta_{q}\sigma_{pr}$$

for all 1≤r≤k. We thus complete the proof.

The conclusions in Theorems 3.2 and 3.3 can easily apply to the original PDE system (2.1) under the constraint (2.3). In each of the following scenarios, the basic reproduction number ${ℛ}_{0}^{\text{PDE}}$ for the reaction-diffusion system (2.1) and the basic reproduction number ${ℛ}_{0}^{\text{ODE}}$ for its ODE counterpart (A.1) are the same. These results cover several special, but important, cases associated with the PDE model (2.1).

**Corollary 3.1.** *If there exist constants* $\delta_{p},1\leq p\leq m$, *and a continuous function* d(x) such that $d_{p}\left(x\right)=\delta_{p}d\left(x\right)$ *for all* p=1, …,m *in system* (2.1), *then* ${ℛ}_{0}^{PDE}={ℛ}_{0}^{ODE}$.

**Corollary 3.2.** (A scenario with constant diffusion rates.) *If the diffusion rates of all the infected compartments are positive constants in system* (2.1), *then* ${ℛ}_{0}^{PDE}={ℛ}_{0}^{ODE}$.

**Corollary 3.3.** (A scenario with uniform diffusion patterns.) *If* $d_{p}\left(x\right)=d_{q}\left(x\right)$ *for all the infected compartments* (1≤p,q≤m) *in system* (2.1), *then* ${ℛ}_{0}^{PDE}={ℛ}_{0}^{ODE}$.

**Corollary 3.4.** (A scenario with partial diffusion.) *If* $d_{p}\left(x\right)=0$ *for* p=1, …,m−1 *and* $d_{m}\left(x\right)\geq d_{0}>0$ *in system* (2.1), *then* ${ℛ}_{0}^{PDE}={ℛ}_{0}^{ODE}$.

## 4. Two examples

Several numerical examples concerned with one-dimensional (1D) reaction-diffusion epidemic models were presented in [24] to demonstrate that they have the same basic reproduction numbers as those of their ODE counterparts. Now we extend the numerical studies to two-dimensional (2D) and three-dimensional (3D) spatial domains to verify some of our analytical predictions in Section 3.

### 4.1. A 2D SIR model

We consider a host population that moves on a 2D spatial domain represented by ${\left[0,1\right]}^{2}$, where the motion can be described by a diffusion process. Let S,I and R be the density of the susceptible, infected, and recovered individuals, respectively, and $d_{S}\left(x\right),d_{I}\left(x\right)$ and $d_{R}\left(x\right)$ be their associated diffusion rates with $x=\left(x_{1},x_{2}\right)\in {\left[0,1\right]}^{2}$. We study the following 2D reaction-diffusion SIR system, which is extended from the model presented in [4]:

(4.1) $$\frac{\partial S}{\partial t}=\nabla \cdot \left(d_{S}\left(x\right)\nabla S\right)+\Lambda -\alpha SI-\mu S,\;x\in {\left[0,1\right]}^{2},\;t>0;\;\frac{\partial I}{\partial t}=\nabla \cdot \left(d_{I}\left(x\right)\nabla I\right)+\alpha SI-\left(\mu +\gamma \right)I,\;x\in {\left[0,1\right]}^{2},\;t>0;\;\frac{\partial R}{\partial t}=\nabla \cdot \left(d_{R}\left(x\right)\nabla R\right)+\gamma I-\mu R,\;x\in {\left[0,1\right]}^{2},\;t>0.$$

The constant parameters Λ,α,μ, and γ denote the recruitment rate, transmission rate, natural death rate, and disease recovery rate, respectively. Disease-induce mortality is not included here. Neumann boundary conditions are imposed on the boundary $\partial {\left[0,1\right]}^{2}$ and appropriate initial conditions are provided at t=0.

Obviously, I is the only infected compartment in system (4.1); i.e., m=1, so that Theorem 3.1(2) applies. From the underlying ODE system, we obtain $F=\frac{\alpha \Lambda }{\mu }$ and V=μ+γ. Then the basic reproduction number of the PDE system (4.1) is the same as that of its corresponding ODE system, based on Theorem 3.1(2):

$${ℛ}_{0}^{\text{PDE}}=\rho \left(FV^{-1}\right)=\frac{\alpha \Lambda }{\mu \left(\mu +\gamma \right)}.$$

**Figure 1.**
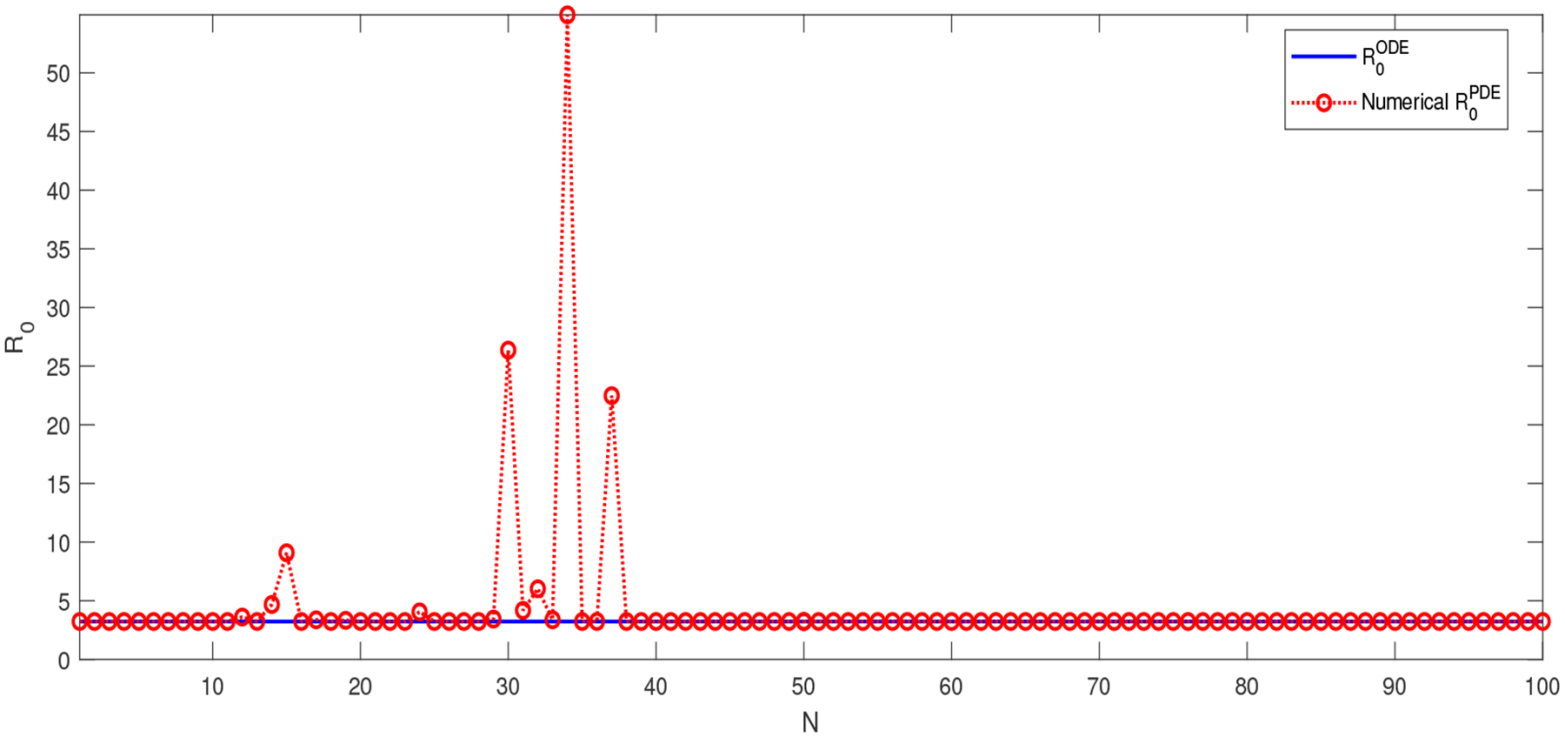
Comparison between ${ℛ}_{0}^{\text{ODE}}$ and ${ℛ}_{0}^{\text{PDE}}$ for the 2D SIR model $\left(4.1\right)\cdot {ℛ}_{0}^{\text{ODE}}\approx 3.20$ is independent of $N\cdot {ℛ}_{0}^{\text{PDE}}$ is numerically calculated by $\rho \left(\left(F⊗I_{{\left(N+1\right)}^{2}}\right)A^{-1}\right)$ for each N.

To verify this relationship, Figure 1 compares ${ℛ}_{0}^{\text{PDE}}$ and ${ℛ}_{0}^{\text{ODE}}$ for this model. ${ℛ}_{0}^{\text{PDE}}$ is computed by our numerical method based on Eq (3.8). The values of $\rho \left(\left(F⊗I_{{\left(N+1\right)}^{2}}\right)A^{-1}\right)=\rho \left(\frac{\alpha \text{Λ}}{\mu }A_{1}^{-1}\right)$ versus N (N=1,2,⋯) are plotted in Figure 1, where $A_{1}$ is the matrix obtained in Eq (3.4) from the single infectious compartment I. Meanwhile, since ${ℛ}_{0}^{\text{ODE}}=\rho \left(FV^{-1}\right)$ does not depend on N, it is represented by a horizontal line in the graph. We set the diffusion rate of the infected individuals as $d_{I}\left(x\right)=\text{sin}\left(100\left(x_{1}+x_{2}\right)\right)+2$ in this test. We observe that when N is sufficiently large, the numerical values of ${ℛ}_{0}^{\text{PDE}}$ based on $\rho \left(\left(F⊗I_{{\left(N+1\right)}^{2}}\right)A^{-1}\right)$ almost perfectly match ${ℛ}_{0}^{\text{ODE}}$, and this pattern continues for all N≥40.

**Figure 2.**
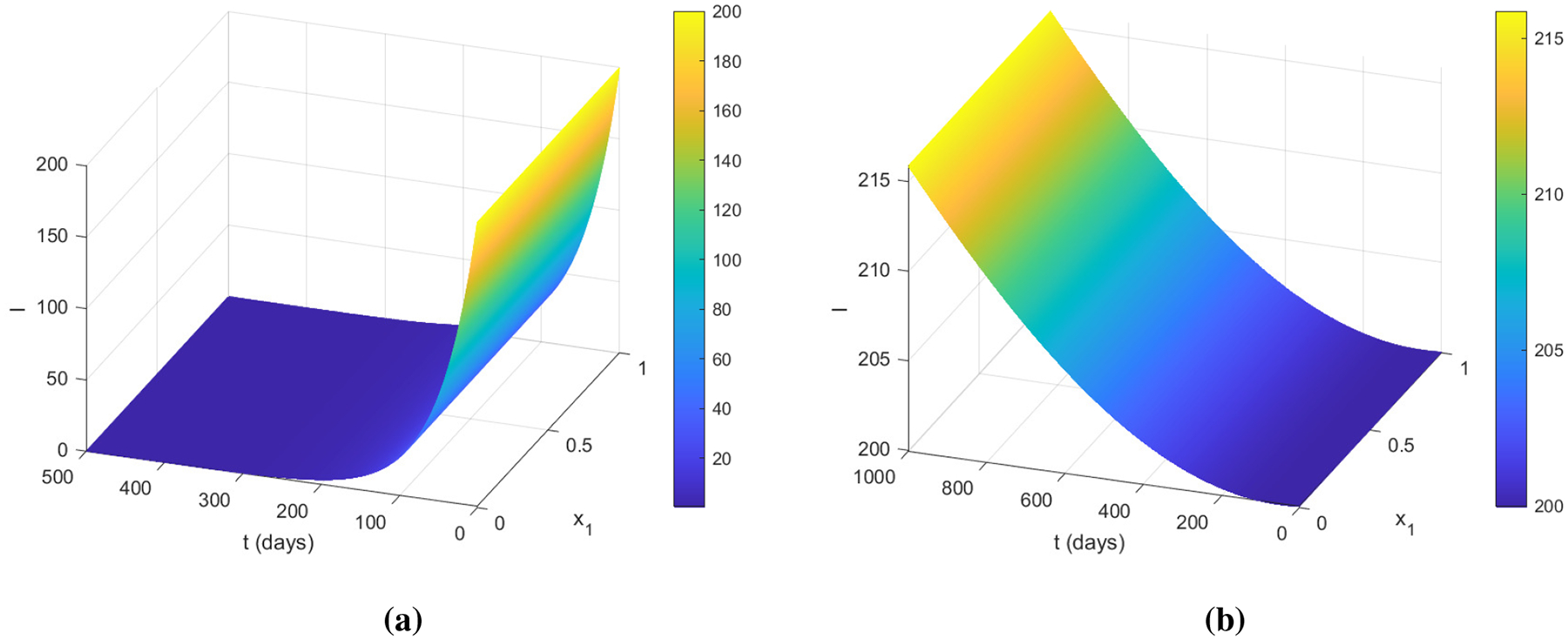
Two typical scenarios of $I\left(t,x_{1},0.5\right)$ versus t and $x_{1}$ for the SIR model (4.1): (a) ${ℛ}_{0}^{\text{PDE}}=0.88$; (b) ${ℛ}_{0}^{\text{PDE}}=3.20$.

Next, we verify that ${ℛ}_{0}^{\text{PDE}}=1$ can be used as a threshold to distinguish the two dynamical behaviors between disease eradication and disease persistence for the model (4.1). To that end, we consider two typical scenarios of ${ℛ}_{0}^{\text{PDE}}<1$ and ${ℛ}_{0}^{\text{PDE}}>1$ by selecting appropriate parameter values, and run the simulation for the model (4.1) with an initial infection density $I\left(0,x_{1},x_{2}\right)=200$ in each scenario. Figure 2(a) displays the surface plot of $I\left(t,x_{1},x_{2}=0.5\right)$ versus t and $x_{1}$ with ${ℛ}_{0}^{\text{PDE}}=0.88<1$, where I approaches 0 over time for all $x_{1}$. In contrast, Figure 2(b) displays the surface plot of $I\left(t,x_{1},x_{2}=0.5\right)$ versus t and $x_{1}$ with ${ℛ}_{0}^{\text{PDE}}=3.20>1$, where I increases from its initial value and remains positive for all the time. Surface plots at other fixed values of $x_{2}$ and those with $x_{1}$ fixed (not shown here) are qualitatively similar.

### 4.2. A 3D model for environmentally transmitted diseases

Environmentally transmitted diseases continue to impose a significant public health burden throughout the world [29]. The transmission dynamics of many such diseases can be studied by SIR-B models [30–32], where the B compartment typically represents the concentration of the pathogen in the contaminated environment. Here, we focus on airborne infections, where the pathogenic particles (especially those tiny particles such as aerosols) can float and move in the air for an extended period of time. These airborne pathogens include bacteria such as Mycobacterium, Staphylococcus, and Legionella [33], viruses such as Varicella, Hantavirus, and SARS-CoV-2 [34], and other microorganisms such as fungi [35].

We consider a reaction-diffusion SIR-B model that incorporates both the indirect (i.e., airborne) and direct (i.e., human-to-human) transmission routes for an airborne infection. We assume that the pathogen undergoes a diffusion process in the air in a 3D domain represented by [0,1]^3^. We also assume that, compared to the pathogen diffusion and dispersal, the average spatial movement of human hosts is slow and can be disregarded in our model. We thus obtain the following PDE system

(4.2) $$\frac{\partial S}{\partial t}=\Lambda -\left(\alpha I+\beta B\right)S-\mu S;\;\frac{\partial I}{\partial t}=\left(\alpha I+\beta B\right)S-\left(\mu +\gamma \right)I;\;\frac{\partial R}{\partial t}=\gamma I-\mu R;\;\frac{\partial B}{\partial t}=\nabla \cdot \left(d_{B}\left(x\right)\nabla B\right)+\xi I+rB\left(1-\frac{B}{K}\right)-\tau B,$$

for t>0 and $x=\left(x_{1},x_{2},x_{3}\right)\in {\left[0,1\right]}^{3}$, with Neumann boundary conditions and appropriate initial conditions. The parameters α and β represent, respectively, the direct and indirect transmission rates, ξ is the rate of contribution from infected individuals (through coughing, sneezing, etc.) to the pathogen in the air, r is the intrinsic growth rate of the pathogen (for viruses, we may set r=0),K is the carrying capacity of the pathogen growth, and τ is the removal rate of the pathogen from the air. A bilinear incidence form is used to represent both the direct and indirect transmission routes.

The model (4.2) is a partially diffusive PDE system since the diffusion component is only incorporated into the pathogen equation. Therefore, Corollary 3.4 predicts that ${ℛ}_{0}^{\text{PDE}}$ of system (4.2) equals ${ℛ}_{0}^{\text{ODE}}$ of the underlying ODE system. The infectious compartments are I and B. From the associated ODE system, we easily obtain

$$F=\left[\begin{array}{cc} \frac{\alpha \Lambda }{\mu } & \frac{\beta \Lambda }{\mu }\\ \xi & r \end{array}\right]\text{ and }V=\left[\begin{array}{cc} \mu +\gamma & 0\\ 0 & \tau \end{array}\right].$$

From Corollary 3.4, we obtain

(4.3) $${ℛ}_{0}^{\text{PDE}}=\rho \left(FV^{-1}\right)=\frac{1}{2}\left(\frac{\alpha \Lambda }{\mu \left(\mu +\gamma \right)}+\frac{r}{\tau }+\sqrt{{\left(\frac{\alpha \Lambda }{\mu \left(\mu +\gamma \right)}-\frac{r}{\tau }\right)}^{2}+\frac{4\xi \beta \Lambda }{\mu \tau \left(\mu +\gamma \right)}}\right).$$

**Figure 3.**
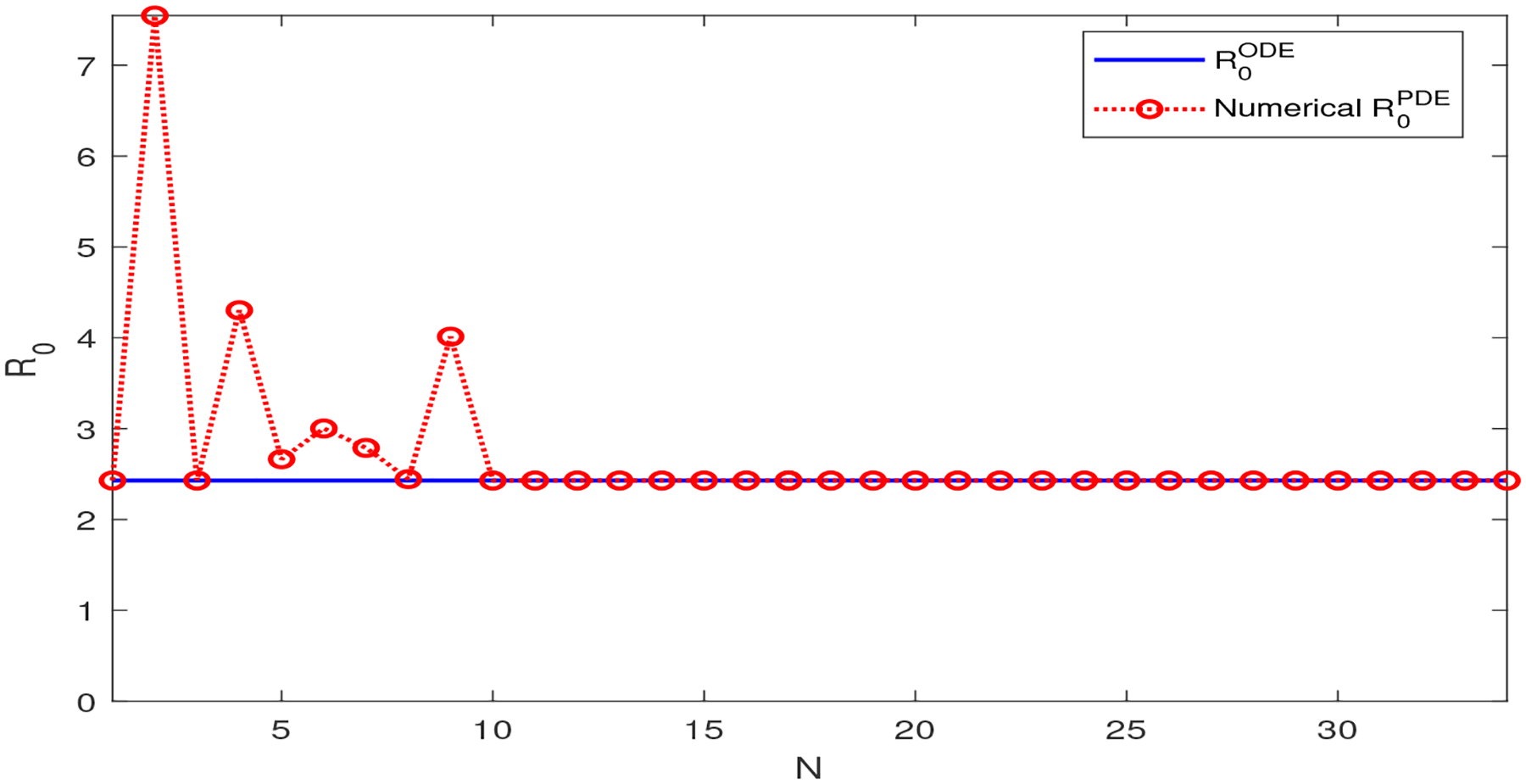
Comparison between ${ℛ}_{0}^{\text{ODE}}$ and ${ℛ}_{0}^{\text{PDE}}$ for the 3D SIR-B model (4.2). ${ℛ}_{0}^{\text{ODE}}\approx 2.43$ is independent of N. ${ℛ}_{0}^{\text{PDE}}$ is numerically calculated by $\rho \left(\left(F⊗I_{{\left(N+1\right)}^{3}}\right)A^{-1}\right)$ for each N.

To provide numerical evidence for this analytical relationship, we compare ${ℛ}_{0}^{\text{PDE}}$ and ${ℛ}_{0}^{\text{ODE}}$ in Figure 3, where we plot $\rho \left(\left(F⊗I_{{\left(N+1\right)}^{3}}\right)A^{-1}\right)$ versus N(N=1,2,⋯) for the model (4.2). Note that $\left(F⊗I_{{\left(N+1\right)}^{3}}\right)A^{-1}=\left(\begin{array}{cc} \frac{\alpha \Lambda }{\mu \left(\mu +\gamma \right)}I_{{\left(N+1\right)}^{3}} & \frac{\beta \Lambda }{\mu }A_{2}^{-1}\\ \frac{\xi }{\mu +\gamma }I_{{\left(N+1\right)}^{3}} & rA_{2}^{-1} \end{array}\right)$ and $A_{2}$ is the matrix obtained in Eq (3.4) from the infectious compartment B. In order to clearly show the convergence of the numerical values of $R_{0}^{\text{PDE}}$, we set the pathogen diffusion rate as $d_{B}\left(x\right)=10^{-3}\left(\text{sin}\left(100\left(x_{1}+x_{2}+x_{3}\right)\right)+1.01\right)$ in this test. We observe a pattern similar to that in Figure 1. Specifically, the numerical approximations of ${ℛ}_{0}^{\text{PDE}}$ based on $\rho \left(\left(F⊗I_{{\left(N+1\right)}^{3}}\right)A^{-1}\right)$ coincide with ${ℛ}_{0}^{\text{ODE}}$ for all N>10.

**Figure 4.**
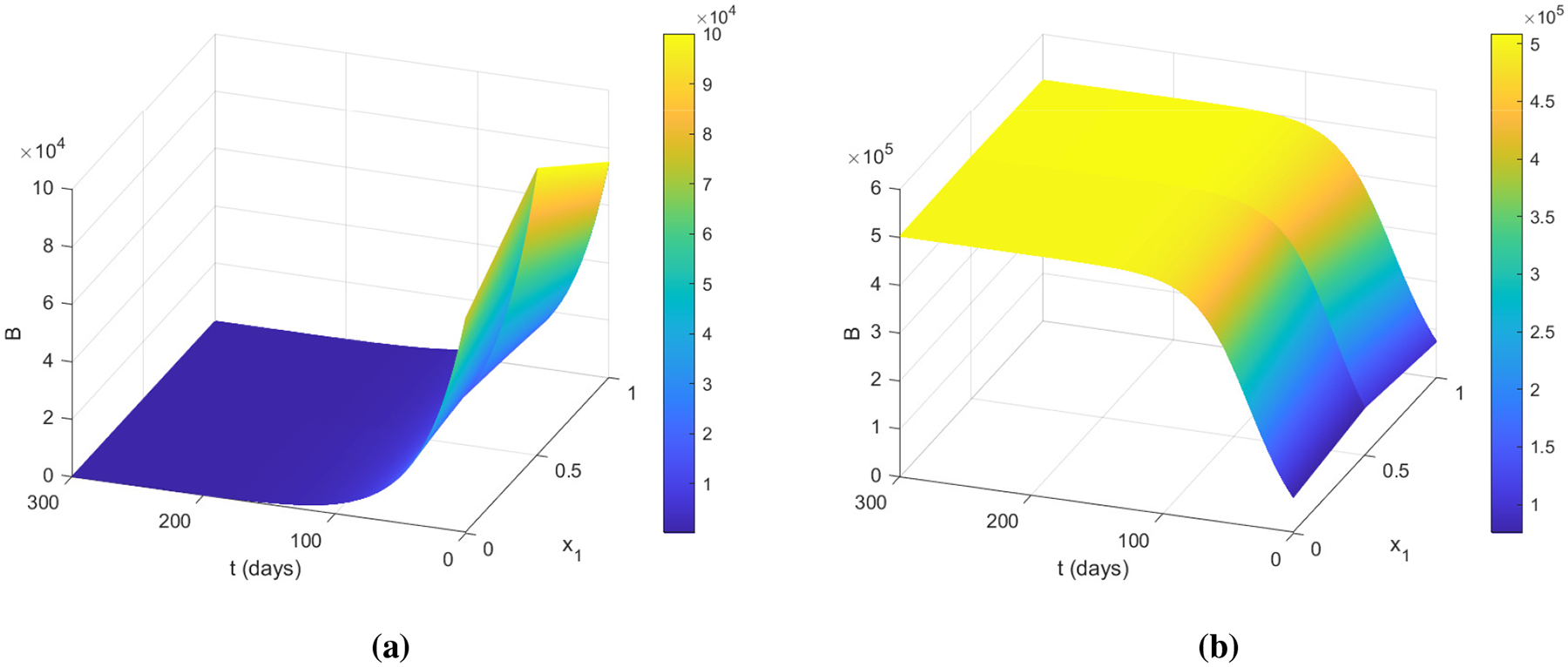
Two typical scenarios of $B\left(t,x_{1},0.5,0.5\right)$ versus t and $x_{1}$ for the SIR-B model (4.2): (a) ${ℛ}_{0}^{\text{PDE}}=0.72$; (b) ${ℛ}_{0}^{\text{PDE}}=2.43$.

The stability properties for a more general partially diffusive SIR-B model were analyzed in [36] and it was shown that ${ℛ}_{0}^{\text{PDE}}=1$ provided a threshold for the transition between disease eradication and disease persistence. We now provide some numerical verification for the PDE model (4.2). We again consider two typical scenarios with ${ℛ}_{0}^{\text{PDE}}<1$ and ${ℛ}_{0}^{\text{PDE}}>1$, and set the initial concentration of the pathogen as $B\left(0,x_{1},x_{2},x_{3}\right)=10^{5}\left(3-{\left(x_{1}-0.5\right)}^{2}-{\left(x_{2}-0.5\right)}^{2}-{\left(x_{3}-0.5\right)}^{2}\right)$ for the model (4.2). We then run the simulation in each scenario and plot the pathogen concentration $B\left(t,x_{1},x_{2}=0.5,x_{3}=0.5\right)$ versus t and $x_{1}$ in Figure 4, where we clearly see the eradication of the pathogen in panel (a) and the persistence of the infection in panel (b). Other surface plots with various values of $x_{1},x_{2}$, and $x_{3}$ have similar behaviors and are not shown here.

## 5. Conclusions

In this paper, we are concerned with a class of reaction-diffusion epidemic models whose underlying ODE systems are autonomous. We have proposed a computational approach to efficiently calculate and analyze the basic reproduction numbers, ${ℛ}_{0}^{\text{PDE}}$, for such PDE models. The present work is an extension of our previous study in [24] from one-dimensional spatial domains to k-dimensional domains for any positive integer k. This extension contributes to a more holistic understanding for the basic reproduction numbers of reaction-diffusion epidemic systems, and allows broader applications of the methodology in epidemiological studies.

Our numerical method transfers the computation of ${ℛ}_{0}^{\text{PDE}}$, defined as the spectral radius of an operator that is infinite-dimensional, to the calculation of the principal eigenvalue associated with a finite-dimensional matrix. Such a formulation enables us to apply the matrix theory to analyze and compare the basic reproduction numbers for the PDE system and its underlying ODE system. We have found that ${ℛ}_{0}^{\text{PDE}}={ℛ}_{0}^{\text{ODE}}$ in several special but important cases, such as (i) a single infected compartment in the system; (ii) constant diffusion rates; (iii) uniform diffusion patterns in the infected compartments; and (iv) the presence of partial diffusion. For these scenarios, the computation of ${ℛ}_{0}^{\text{PDE}}$ can be conveniently handled by using ${ℛ}_{0}^{\text{ODE}}$, saving unnecessary efforts in the operator and eigenvalue analysis associated with the PDE models.

Prior studies on the basic reproduction numbers of reaction-diffusion epidemic models are often focused on the analysis of the asymptotic profiles when the constant diffusion rates tend to zero or infinity (e.g., [13–16]). In contrast, our work aims to explore a more general relationship between the basic reproduction numbers of the PDE models with variable diffusion rates and those of their underlying autonomous ODE models. Another unique feature of our study is that the analytical work is inspired by the numerical formulation and involves only elementary numerical techniques and matrix theory. The findings in this study help to efficiently quantify the risk of disease transmission for a large class of reaction-diffusion models. We hope to extend the methodology to reaction-convectiondiffusion systems and possibly other PDE epidemic models in our future research.

## Appendix

### A. Underlying ODE model

If system (2.1) is homogeneous in space; i.e., $U={\left(u_{1}\left(t\right),\ldots ,u_{n}\left(t\right)\right)}^{T}$, then the PDE model (2.1) is reduced to the following ODE model

(A.1)
$$\frac{dU}{dt}=ℱ\left(U\right)-𝒱\left(U\right),\;t>0,$$

where $ℱ\left(U\right)={\left({ℱ}_{1}\left(U\right),\;\ldots ,{ℱ}_{n}\left(U\right)\right)}^{T}$ and $𝒱\left(U\right)={𝒱}^{-}\left(U\right)-{𝒱}^{+}\left(U\right)={\left({𝒱}_{1}\left(U\right),\;\ldots ,{𝒱}_{n}\left(U\right)\right)}^{T}$. Based on the framework in [10] for ODE epidemic models, we make the following standard assumptions:

(A1) ${ℱ}_{i}\left(U\right),{𝒱}_{i}^{+}\left(U\right),{𝒱}_{i}^{-}\left(U\right)$ are non-negative and continuously differentiable, 1≤i≤n.

(A2) If $u_{i}=0$, then ${𝒱}_{i}^{-}=0,1\leq i\leq m$.

(A3) ${ℱ}_{i}=0$ for i>m.

(A4) If $U\in U_{s}$, then ${ℱ}_{i}={𝒱}_{i}^{+}=0,i=1,\;\ldots ,m$.

Suppose that $U^{0}=\left(0,\;\ldots ,0,u_{m+1}^{0},\;\ldots ,u_{n}^{0}\right)$ is a disease-free steady state of model (A.1) which is spatially independent. Then the basic reproduction number for the ODE system (A.1) is defined as the spectral radius of the next-generation matrix [10]; i.e.,

(A.2)
$${ℛ}_{0}^{\text{ODE}}=\rho \left(FV^{-1}\right),$$

where F and V are m×m constant matrices with (i,j) entry $F_{ij}=\frac{\partial {ℱ}_{i}}{\partial u_{j}}\left(U^{0}\right)$ and $V_{ij}=\frac{\partial {𝒱}_{i}}{\partial u_{j}}\left(U^{0}\right)$, respectively.

## B. Next-generation operator

For t>0 and any solution ϕ(t,x) of Eq (2.4),

(B.1)
$$\underset{s\to 0+}{\text{lim}}\frac{T\left(s\right)\phi \left(t,x\right)-\phi \left(t,x\right)}{s}=\underset{s\to 0+}{\text{lim}}\frac{\phi \left(t+s,x\right)-\phi \left(t,x\right)}{s}=\frac{\partial \phi }{\partial t}\left(t,x\right)=\Gamma \left(\phi \right).$$

Hence, Γ is the generator of the $C_{0}$-semigroup T(t) on $C\left({\left[0,1\right]}^{k},{\mathbb{R}}^{m}\right)$. Note that T(t) is a positive semigroup since $T\left(t\right)C\left({\left[0,1\right]}^{k},{\mathbb{R}}_{+}^{m}\right)\subset C\left({\left[0,1\right]}^{k},{\mathbb{R}}_{+}^{m}\right)$ for all t≥0. Let σ(Γ) denote the spectrum of the operator Γ. It then follows from Theorem 3.12 in [11] that

(B.2)
$${\left(\lambda I-\Gamma \right)}^{-1}\left(\phi \right)=\int_{0}^{+\infty }e^{\lambda t}T\left(t\right)\left(\phi \right)dt,\;\forall \lambda >s\left(\Gamma \right),\phi \in C\left({\left[0,1\right]}^{k},{\mathbb{R}}^{m}\right),$$

where s(Γ)=sup{Reλ:λ∈σ(Γ)} is the spectral bound of Γ. Due to the loss of infected individuals from natural and disease-induced mortalities, the internal evolution of individuals in the infectious compartments is usually dissipative and exponentially decaying. We thus assume (B1) −V is cooperative and s(Γ)<0.

Using assumption (B1) and setting λ=0 in Eq (B.2), we obtain

(B.3)
$$L\left(\phi \right)=F\int_{0}^{+\infty }T\left(t\right)\left(\phi \right)dt=-F\Gamma^{-1}\left(\phi \right),$$

or $L=-F{\text{Γ}}^{-1}$. As done in [24], we make two additional assumptions regarding the PDE system (2.1) and its underlying ODE system (A.1):

(B2) There exists a constant $d_{0}$ such that $d_{i}\left(x\right)\geq d_{0}>0$ for any $x\in {\left[0,1\right]}^{k}$ and 1≤i≤m.

(B3) $V=\text{diag}\left(v_{1},\;\ldots ,v_{m}\right)$ with $v_{i}>0,i=1,\;\ldots ,m$.

Condition (B2) sets a minimal diffusion rate at all spatial locations for the PDE model. Condition (B3) is satisfied by many common epidemic models. In case V is not a diagonal matrix, it is often possible to introduce a new infection vector, $\tilde{ℱ}\left(U\right)$, and a new transfer vector, $\tilde{𝒱}\left(U\right)$, in the ODE system (A.1) such that $\frac{dU}{dt}=ℱ-𝒱=\tilde{ℱ}-\tilde{𝒱}$, where the vectors $\tilde{ℱ}$ and $\tilde{𝒱}$ produce a new generation matrix $\tilde{F}$ and a diagonal transitive matrix $\tilde{V}$, respectively. From [10], we know that $\rho \left(FV^{-1}\right)$ and $\rho \left({\tilde{FV}}^{-1}\right)$ are equivalent in characterizing the disease threshold in the sense that they are simultaneously higher than (or lower than) unity.
